# Supplementary material for: Osteopontin Predicts Three-Month Outcome in Stroke Patients Treated by Reperfusion Therapies
Source: J Clin Med. 2020 Dec 13;9(12):4028. doi: 10.3390/jcm9124028 (PMC7763291; doi:10.3390/jcm9124028)
Supplement: Supplementary file 1 [file jcm-09-04028-s001.pdf]

Table S1. Patient's and treatment characteristics in the included and non-included patients.

Characteristics	Included		ASD
	**No (n = 467)**	**Yes (n = 151)**	
Age, median (IQR)	74 (60 to 84)	73 (58 to 83)	8.7
Men	250/467 (53.5)	78/151 (51.7)	3.8
Medical history			
Hypertension	286/462 (61.9)	91/149 (61.1)	1.7
Hypercholesterolemia	164/458 (35.8)	57/147 (38.8)	6.1
Diabetes	76/460 (16.5)	11/147 (7.5)	28.1
Current smoking	78/436 (17.9)	28/144 (19.4)	4.0
Antithrombotic medications	188/463 (40.6)	51/148 (34.5)	12.7
Antiplatelet	151/463 (32.6)	39/148 (26.4)	13.8
Anticoagulant	42/463 (9.1)	14/148 (9.5)	1.3
Pre-stroke mRs >1	141/466 (30.3)	46/149 (30.9)	1.3
Current stroke event			
Admission Systolic BP, mmHg, mean (SD) [1]	154 (22)	154 (20)	1.6
Admission Diastolic BP, mmHg, mean (SD) [1]	81 (14)	81 (13)	3.3
Admission Glucose, mg/dl, mean (SD) [2]	135 (50)	128 (38)	15.6
Admission Platelet counts, mean (SD) [3]	227 (71)	226 (74)	2.3
Admission NIHSS, median (IQR)	9 (4 to 16)	13 (5 to 18)	24.2
Documented arterial occlusion [6]			
None	252/465 (54.2)	46/149 (30.9)	50.1
ICA isolated or tandem with MCA	49/465 (10.5)	35/149 (23.5)	
MCA isolated	153/465 (32.9)	65/149 (43.6)	
Posterior circulation	11/465 (2.4)	3/149 (2.0)	
Cardioembolic etiology	221/456 (48.5)	72/149 (48.3)	0.3
Treatment characteristics			
AIS treatment			
IV thrombolysis	305/467 (65.3)	68/151 (45.0)	41.0
Combined IV thrombolysis and Endovascular treatment(EVT)–	107/467 (22.9)	55/151 (36.4)	
EVT alone	55/467 (11.8)	28/151 (18.5)	
Mechanical endovascular treatment (MET)	64/467 (13.7)	54/151 (35.8)	52.9
Onset to treatment time, min, median (IQR)			
IVT [1]	170 (120 to 194)	152 (120 to 194)	23.6
Combined IVT–EVT [4]	135 (107 to 180)	133 (104 to 152)	19.7
EVT alone [5]	300 (210 to 350)	315 (239 to 350)	12.7

Values are number (%) unless otherwise as indicated. [1] 5 missing values; [2] 15 missing values; [3] 8 missing values; [4] 1 missing value, [5] 3 missing values. [6] documented before AIS treatment by transcranial Doppler, MRA or CT angiography. Abbreviations: AIS = acute ischemic stroke; BP = blood pressure; CT = computed tomography; EVT = endovascular treatment; ICA = internal carotid artery; IQR = interquartile range; IVT = intravenous thrombolyis; MCA = middle cerebral artery; MET = mechanical treatment; MRA = magnetic resonance angiography; NIHSS = National Institutes of Health Stroke Scale; SD = standard deviation.

Table S2. Distribution of biological markers in the included patients.

Blood Biomarkers, in ng/mL	n	Values
Hemostasis		
uPA/urokinase	151	1.2 (1.0 to 1.3)
Serpin E1/PAI-1	151	40 (21 to 66)
Serpin C1/antithrombin-III	150	4546 (2897 to 5708)
Kallikrein 6/Neurosin	150	4.8 (3.8 to 5.8)
Alpha2 macroglobulin	151	2474 (946)
Inflammation		

MPO	151	42 (31 to 62)
CCL2/MCP-1	150	0.25 (0.18 to 0.31)
Adiponectine	150	7752 (5175 to 13519)
Resistin	145	8.8 (6.6 to 11.5)
cDNA	151	0.97 (0.90 to 1.10)
CD40 Ligand	144	0.89 (0.64 to 1.47)
Endothelial activation		
VCAM-1	147	972 (705 to 1535)
ICAM-1	149	283 (198 to 466)
CD31/PECAM	151	9.7 (8.2 to 11.7)
Tissue remodeling		
Total cathepsin S	151	5.2 (3.8 to 7.6)
Osteopontin	151	31 (22 to 46)
Cystain C	151	779 (660 to 967)
Neuropilin-1	151	215 (175 to 255)
MMP-2	151	243 (192 to 287)
MMP-3	150	8.8 (6.2 to 11.4)
MMP-9	151	135 (64 to 226)
MMP-13	118	0.36 (0.29 to 0.42)

Values are median (IQR) except for Alpha2 macroglobulin, where mean (SD) is reported.
